# Recommendations for Imaging of the Temporomandibular Joint. Position Statement from the American Academy of Oral and Maxillofacial Radiology and the American Academy of Orofacial Pain

**DOI:** 10.11607/ofph.3268

**Published:** 2023-03-30

**Authors:** Sanjay M. Mallya, Mansur Ahmad, Joseph R. Cohen, Ghabi Kaspo, Aruna Ramesh

**Affiliations:** ^1^Section of Oral and Maxillofacial Radiology, University of California Los Angeles School of Dentistry, Los Angeles, California, USA; ^2^American Academy of Oral and Maxillofacial Radiology, Lombard, Illinois, USA; ^3^Department of Diagnostic and Biological Sciences, University of Minnesota School of Dentistry, Minneapolis, Minnesota, USA; ^4^Section of Orofacial Pain, AT Still University, Mesa Campus, Mesa, Arizona, USA; ^5^Section of Orofacial Pain, University of California Los Angeles School of Dentistry, Los Angeles, California, USA; ^6^American Academy of Orofacial Pain, Oceanville, New Jersey, USA; ^7^Department of Psychiatry, School of Medicine, Wayne State University, Detroit, Michigan, USA; ^8^Division of Oral and Maxillofacial Radiology, Tufts University School of Dental Medicine, Boston, Massachusetts, USA

**Keywords:** TMJ, Position statement, Recommendations, Imaging examinations

## Abstract

This position statement was developed by an *ad hoc* committee of the American Academy of Oral and Maxillofacial Radiology and the American Academy of Orofacial Pain. The committee reviewed the pertinent literature and drafted recommendations for imaging. This joint statement provides evidence-based recommendations and clinical guidance for applying appropriate diagnostic imaging to evaluate the temporomandibular joint (TMJ). This manuscript guides the design of TMJ imaging examinations, addresses in-office CBCT imaging, and provides timely evidence-based recommendations to evaluate the TMJ bony components, also addressing the use of MRI and other modalities to evaluate TMJ involvement in different pathologic conditions.

There are multiple approaches to diagnosing diseases of the dentomaxillofacial complex. Visual observation may be sufficient to establish a diagnosis for certain conditions (*e.g.*, occlusal caries and periodontal diseases), but histopathologic examinations typically serve as the gold standard for diagnosing pathoses such as cysts and tumors. In contrast, temporomandibular disorders (TMDs) pose a serious diagnostic challenge, as visual examinations provide little information and histopathologic examinations are aggressively invasive with collateral tissue morbidity. Thorough clinical examination and radiologic imaging are the primary diagnostic tools used to identify etiologic contributors and to define the extent of diseases of the TMJ. To optimally harvest the diagnostic information, clinicians should know the capabilities and limitations of the available imaging techniques.

This position statement from the American Academy of Oral and Maxillofacial Radiology (AAOMR) and the American Academy of Orofacial Pain (AAOP) serves as a guide for clinicians to design appropriate radiologic examinations to diagnose and manage TMDs, with a specific emphasis on the indications for CBCT. This statement considers the application of technologies including computed tomography (CT) and magnetic resonance imaging (MRI) for diagnostic evaluation of the TMJ and incorporates recommendations for TMJ imaging. This position statement replaces a position paper on imaging of the TMJ previously published by the AAOMR [1].

The term TMDs includes a group of musculoskeletal and neuromuscular conditions that involve the TMJs, masticatory muscles, and all associated tissues [2]. TMDs are a major cause of nonodontogenic pain in the maxillofacial region. These heterogenous disorders are most often multifactorial in etiology and can affect the masticatory muscles and the bony and soft tissue TMJ components, including the articular disc and its attachments. It has been estimated that 4.8% to 42.7% of the general population manifest some signs or symptoms of TMDs [3, 4]. The symptoms of TMDs and orofacial pain place an economic burden of approximately 4 billion dollars annually on the US health care system [4].

The AAOP classification divides orofacial pain into seven categories [5]. In this classification scheme, TMDs comprise one category of orofacial pain disorders, which is further divided into two broad groups: TMJ disorders and masticatory muscle disorders. The initial diagnostic classification of TMDs is based on the presenting symptoms and clinical examination. However, clinical examination cannot completely assess the osseous and soft tissue components of the TMJ, which present important determinants of the disease category. Imaging findings often corroborate the clinical impressions and help confirm the clinical diagnosis, but imaging may detect pathologic changes that were not clinically detectable and provide information that influences the treatment plan. Thus, appropriate application of imaging is an important element in the diagnosis and treatment planning of an orofacial pain/TMD patient.

## Imaging of TMDs

In general, the diagnostic objectives of imaging a patient with suspected TMDs and associated comorbidities are the following:

1. Evaluate the osseous and soft tissue components of the TMJ.

A. Evaluate the morphology and integrity of the condyle, glenoid fossa, and articular eminence.

B. Evaluate the morphology of the articular disc and its attachments and tissues within the joint capsule.

C. Assess the anatomical and functional relationships between the condyle, articular disc, and glenoid fossa.

2. Evaluate the dentomaxillofacial region to identify coexisting diseases and conditions that may contribute to the patient’s symptoms.

3. Evaluate the dentomaxillofacial region to identify structural and functional consequences of TMDs.

4. Monitor treatment outcomes.

### Imaging Techniques

Current modalities for TMJ imaging include panoramic radiography, CBCT, CT, and MRI. A nuclear medicine bone scan can provide functional information regarding the metabolic status of tissues and may have applications for assessing specific TMD patients.

#### Intraoral imaging

Intraoral imaging is of limited value for a patient with suspected TMDs, but can be useful for identifying sources of odontogenic orofacial pain and is used as an adjunct to evaluate dentate and edentulous regions that are suspected to be potential causes of the symptoms. Pain from dental disease often mimics TMD; therefore, comprehensive evaluation of intraoral radiographs is important to identify potential sources of referred pain, such as pericoronal inflammation around a mandibular third molar or areas of periapical inflammation. Notably, there are no definite relationships between the number of teeth and periodontal bone loss or TMDs [6].

#### Panoramic imaging

Panoramic radiographs can serve as the initial examination for patients with TMD symptoms. Such symptoms are often odontogenic in origin; *e.g.*, an impacted third molar or periapical inflammation. An optimally exposed panoramic radiograph can depict condylar morphology and identify anatomical variants of relevance. However, panoramic imaging has several limitations when it comes to TMJ assessment—for instance, the anterior surface of the condyle, a common location for osteophytes, is not adequately recorded on a panoramic radiograph [7], and the shape of the condyle is often distorted [8, 9]. The zygomatic process of the temporal bone and the articular eminence are frequently superimposed over the condyle, obscuring osseous changes.

#### Extraoral imaging

Classical TMJ projections, such as transcranial, transpharyngeal, and transorbital radiographs, are not currently recommended for evaluation of the TMJ. However, frontal and lateral cephalometric radiographs may have ancillary value in monitoring the consequences of TMDs. Cephalometric imaging can provide information on facial asymmetry resulting from growth disturbances of the condyle(s) and/or treatment-associated changes with orthotic, orthodontic, or orthopedic appliances, as relevant to TMD evaluation [10]. Importantly, cephalometric imaging should not serve as the primary examination to evaluate the TMJ in these clinical situations.

#### Computed tomography

Depending on the configuration of the beam and the detector, CT scanners may be categorized as CBCT or multidetector CT (MDCT). This position statement emphasizes the application of CBCT imaging for the appropriate diagnosis and management of TMDs. Maxillofacial CBCT provides an assessment of the osseous and dental hard tissue components [11, 12]. However, due to its limited soft tissue contrast resolution, CBCT cannot be used to assess soft tissue details, which is information that may be needed to diagnose and manage TMD patients.

Specific considerations to optimize the diagnostic yield from TMJ CBCT examinations are discussed below.

• The field of view (FOV) is a principal parameter that must be optimized for individual CBCT examinations. At minimum, the FOV should encompass the condyle, glenoid fossa, and articular eminence.

• Given that many TMJ afflictions are bilateral, both TMJs should be imaged, either in the same FOV or with independent examinations. If additional coverage of the arches, paranasal sinuses, and/or cervical spine is required for diagnostic assessment, the clinician may select a wider FOV to encompass more anatomy.

• In general, images reconstructed at smaller voxel sizes yield higher spatial resolution. The voxel sizes used in CBCT do not significantly impact diagnostic efficacy for the detection of osseous degenerative changes [13].

• Typically, when imaging the TMJs, the scan should be acquired with the teeth in maximum intercuspation. Open-mouth TMJ imaging has limited impact on TMD diagnosis and management, affecting diagnosis in 3% to 7% of cases and management in 1% to 8% of cases [14]. Open-mouth views may have some role in evaluating limited mouth opening or chronic subluxation.

Both MDCT and CBCT provide excellent imaging of bony TMJ components, with equivalent diagnostic efficacies for the detection of osseous abnormalities. MDCT provides better contrast of the soft tissues and allows for the assessment of muscles and tissue spaces. However, the articular disc is not well depicted on CT and is thus of limited value for evaluating TMJ internal derangements.

#### Magnetic resonance imaging

MRI is the only imaging technique that reliably shows the location of the articular disc. MRI provides cross-sectional imaging of the joints similar to CBCT and MDCT, but also provides valuable information about the soft tissue components of the joint, including the shape, location, and size of the articular disc and the presence of fluid effusion. However, the osseous details are comparatively poor on MRI [15, 16], and current MRI technology cannot fully replace CBCT or MDCT for osseous assessment. Nevertheless, soft tissue details are superior on MRI compared to MDCT and vastly superior compared to CBCT [17].

## Recommendations for Orofacial Pain Arising from Odontogenic Sources

Pathoses of the dental and periapical areas often mimic pain associated with the TMJs. Odontogenic sources account for an estimated 75% of orofacial pain [18]. Periapical inflammation of the maxillary posterior teeth and oroantral fistulas after extraction can lead to sinusitis of odontogenic origin [19, 20]. Pulpal exposure from carious lesions, root resorption, or root fracture can lead to symptoms that could be mistaken for TMDs [21].

### Recommendation 1

Intraoral radiographs/panoramic radiographs should be used to evaluate patients with suspected odontogenic pain. In complex odontogenic pathoses, a limited FOV CBCT scan may be required.

#### Rationale

Conventional imaging using periapical and panoramic radiographs is a low–radiation dose procedure that serves as the first line of radiologic imaging and is often adequate to diagnose and manage patients with suspected odontogenic pathoses. Depending on the clinical situation, the clinician may opt to selectively image regions with periapical radiographs or use full-mouth intraoral and panoramic imaging when the symptoms and findings are more generalized. However, the two-dimensional nature of these images limits their ability to detect vertical and horizontal root fractures [22], root resorptions [23], and vertical root fractures [24]. The American Association of Endodontists and the American Academy of Oral and Maxillofacial Radiology have published a position statement that outlines the use of CBCT imaging for endodontic diagnosis and treatment planning [25]. Many of the situations described previously would be served by the guidance in that position statement for appropriate selection of imaging.

#### Recommendations for arthritic diseases of the TMJ

Arthritic diseases include cartilage degradation by chronic deterioration (osteoarthritis), circulating autoantibodies (systemic arthritides), or unknown causes (idiopathic condylar resorption). TMJ arthritis is rarely infective in nature; rather, it is typically either systemic or traumatic in origin. The symptom history and clinical examination provide the clinician with adequate clues as to the etiologic nature of the presenting symptoms to make a provisional diagnosis. These arthritic conditions primarily affect osseous components of the TMJ complex:

1. Degenerative joint disease (DJD, osteoarthritis) results from chronic deterioration of articular tissue. Radiologic findings of DJD include osteophytes, erosions, and subchondral cysts. Flattening and subcortical sclerosis of the articular surfaces are considered signs of remodeling of the joints. The diagnostic criteria for flattening, subcortical sclerosis, osteophytes, erosions, and subchondral cysts have been thoroughly described [26].

2. Systemic arthritides are a group of systemic diseases that cause joint inflammation. The conditions that are frequently encountered in this group include TMD manifestations in patients with rheumatoid arthritis (RA), psoriatic arthritis, juvenile idiopathic arthritis, and chondrocalcinosis. Radiologically, the manifestations of systemic arthritides are similar to changes that occur in degenerative joint disease and include erosions on the condyle/fossa (30% to 86%), flattening of the condyle/fossa (80% to 89%), osteophytes (32% to 65%), and subchondral cysts (32% to 65%) [27, 28, 29, 30, 31, 32, 33]. Importantly, there is no correlation between rheumatoid factor measurements and the severity of TMJ osseous changes, emphasizing the need to independently investigate TMJ involvement with imaging in these patients.

3. Idiopathic condylar resorption (ICR), also termed progressive condylar resorption, is a specific type of condylar resorption that is discrete from DJD and systemic arthritides. The most common radiologic findings of ICR are condylar resorption and volume loss, anterior open bite, a high mandibular plane angle, and decreased ramal height [34, 35, 36]. As with DJD and systemic arthritides, radiographic demonstration of osseous changes (*i.e.*, condylar resorption) is necessary to establish a diagnosis of ICR.

4. Synovial chondromatosis (SC) is characterized by abnormal proliferation and cartilage production by the synovium. Primary SC occurs spontaneously and is of unknown etiology.

5. Secondary SC occurs subsequent to joint inflammations, including those caused by trauma and DJD. There is considerable overlap of the populations affected by SC and the other TMJ arthritic disorders described previously. The common presenting symptoms—joint pain, joint swelling, and limitation of function— are nonspecific and similar to the clinical manifestations of other joint inflammations [37, 38]. The cartilage nodules present as sclerotic or hyperostotic areas attached to or discrete from the glenoid fossa, articular eminence, and mandibular condyle, or as “loose bodies” adjacent to the condyle and temporal bone [38, 39, 40].

### Recommendation 2

CT imaging, preferably CBCT, should be used to evaluate osseous changes in patients with suspected TMJ arthritis. Panoramic radiography has a limited role in establishing arthritic changes.

#### Rationale

Validated clinical diagnostic criteria for TMDs have low sensitivity (55%) and low specificity (61%) in identifying patients with DJD [2]. The sensitivity of panoramic radiographs to detect TMJ osseous changes is low [15, 41, 42, 43]. Moreover, reader agreement for identifying the presence of degenerative changes on panoramic radiographs is poor [15]. Thus, panoramic images are of limited value for critical radiologic evaluation of osteoarthritis. Therefore, the identification of bony changes with CT is necessary to conclusively establish a diagnosis of arthritis. Moreover, the pattern and temporal sequence of osseous changes provide additional clues as to the biologic nature of the arthritic changes and help direct additional investigations and appropriate management. Most *in vivo* studies that examine the efficacy of imaging to assess osseous TMJ changes use CBCT/MDCT as the reference standard, emphasizing the superiority of CBCT/MDCT for evaluating TMJ osseous changes. This information is required to establish the diagnosis of DJD and to direct appropriate management. Panoramic radiography will underdiagnose arthritis due to its low sensitivity, and a false negative imaging result will result in erroneous categorization of the patient’s disease and inappropriate management. This emphasizes the need for CT to better evaluate patients with suspected arthritic changes. Although both MDCT and CBCT are equivalent for osseous assessments, CBCT exposes the patient to lower amounts of radiation and is thus the preferred modality.

#### Diagnosis of osteophytes

In comparison to CT imaging, panoramic radiography has a low sensitivity (12%) for the detection of osteophytes. Notably, the entire condylar surface is not clearly depicted on panoramic radiographs due to the angle of projection, and superimpositions can obscure the presence of osteophytes on the condyle and fossa. CT imaging overcomes these disadvantages, increasing the efficacy of detection [44].

#### Diagnosis of erosions

The extent of erosions may vary, from small surface erosions to larger areas of bony destruction of the articular surface. *In vitro* studies on human skulls have shown that the accuracy of CBCT for detecting cortical erosions is considerably higher than panoramic radiography [43]. Panoramic imaging has a low sensitivity for detecting erosions (20%) and subcortical cysts (14%), underscoring the need for CT imaging [44].

#### Diagnosis of subchondral pseudocysts

As the area of erosion enlarges, it produces a subcortical radiolucency, often referred to as a subchondral “cyst” or subchondral pseudocyst. CT images are superior to panoramic radiography for the diagnosis of subchondral pseudocysts [44, 45].

#### Diagnosis of synovial chondromatosis

Panoramic radiography can demonstrate calcified bodies if located in the anterior joint space. CBCT and MDCT scans can also show loose calcified bodies in the superior joint space, as well as on the lateral and medial aspects of the condyle. The sensitivity of CT for detection of SC is approximately 13%, much lower than the 94% sensitivity of MRI. MRI has a higher detection rate because it identifies noncalcified free bodies. If noncalcified bodies are suspected, clinicians should consider additional MRI to evaluate the TMJ [37, 38].

#### Diagnosis of subcortical sclerosis and articular surface flattening

The sensitivity for detection of sclerosis on panoramic images is only 33%, reinforcing the need for CT imaging [44].

## Recommendation for the Assessment of Developmental Disorders: Condylar Aplasia, Hypoplasia, Condylar Hyperplasia, and Coronoid Hyperplasia

Acquired or developmental changes of the condylar or coronoid processes can be isolated occurrences or associated with craniofacial syndromes. Hypoplasia or hyperplasia of the condyle may be associated with deformity of the ramus or body of the mandible. Acquired benign and malignant neoplasia may also result in enlargement of the condyle, mimicking hyperplasia. Imaging of the joints is needed for diagnosis, prediction of prognosis, and presurgical and postoperative planning [46]. Not all types of congenital deformity of the condyle require surgical correction.

### Recommendation 3

Panoramic radiography should be used for the initial radiologic assessment of a suspected TMJ developmental disorder. CBCT imaging should be acquired for the pre- and postoperative analysis of the TMJ complex.

#### Rationale

Although panoramic radiographs are limited by distortion and frequent positioning errors, they are adequate for identifying developmental disorders of the TMJs, including aplasia, hypoplasia, and hyperplasia. In addition, panoramic imaging provides valuable information on the shape and size of the coronoid process. Often, no other imaging is needed if no surgical correction is planned. Because these developmental disorders are primarily osseous, CBCT can provide detailed information on the osseous morphology and relationships of the TMJ anatomical complex. When surgical correction is planned, quantitative analysis of the mandible and condyles should be obtained from MDCT or CBCT scans [47, 48]. The required quantitative analysis cannot be obtained from panoramic or cephalometric radiography.

## Recommendation for Assessment of Internal Derangement

Displacement of the articular disc can manifest with reduction, with intermittent locking, without reduction or closed lock, or with disc perforation. The classification of internal derangement was described by Wilkes using MRI and complex motion tomography [49].

Based on the DC/TMD data, a new classification of disc displacement with recommendations for imaging protocols was proposed [26]. The TMJ symptoms (joint pain, function, and disability) do not correlate with radiologic findings of disc displacement [50].

### Recommendation 4

Proton density or T1-weighted MRI (PDWI or T1WI) of the TMJ, acquired in the closed- and open-mouth positions, should be used to evaluate patients with suspected disc displacement. When joint space effusion is suspected, T2-weighted MRI (T2WI) should be acquired.

#### Rationale

Clinical assessment (*e.g.*, auditory signals of clicking or popping sounds) characterizes disc displacement, but has low sensitivity in diagnosing displacement [2] and is inadequate for evaluating the status of the internal components of the joint [51]. Narrow joint spaces or a posterior position of the condyle, as observed on MDCT or CBCT, are often considered an indirect assumption of disc displacement. However, MDCT or CBCT examinations do not depict the location or shape of the disc. Arthroscopic examinations add information for identification of disc displacement but are invasive. The current noninvasive examination of choice is PDWI or T1WI MRI in the closed- and open-mouth positions, imaged in the corrected sagittal and corrected coronal planes through the long axis of the condyle [26, 52]. Along with disc displacement, fluid effusion in the joint spaces may contribute to TMD. T2WI MRI is ideal for evaluating fluid accumulation, and effusion is demonstrated with a high signal intensity [53]. Neither MDCT nor CBCT has the ability to demonstrate the presence of effusion.

## Recommendations for Assessment of Trauma

Fractures of the condylar head and neck account for approximately 27% to 42% of mandibular fractures [54] and occur bilaterally in approximately 20% of patients [55]. Condylar fractures can be classified based on location (intracapsular, extracapsular, or subcondylar), displacement (nondisplaced, deviated, or displaced in any orientation), and/or orientation of the fracture (vertical, horizontal, or compression type). The clinical indicators of condylar head/neck fractures include an open bite on the contralateral side, premature occlusion on the ipsilateral side, and deviation on opening to the affected side. When the fracture is bilateral, the patient may develop an anterior open bite with occlusion of the posterior dentition. A patient with a suspected condylar fracture may also have other fractures of the craniofacial complex. The goal of management is to restore occlusion and function. Most condylar fractures are managed with closed reduction. Indications for open reduction include fractures of the adjacent cranial base or external auditory meatus, unstable and comminuted fractures, and inability to establish occlusion. The diagnostic objective of imaging is to detect and characterize the fracture(s), determine and quantify displacement, and evaluate the teeth and dentomaxillofacial skeleton for other fractures.

### Recommendation 5

Panoramic radiography can be used as the initial imaging examination of a patient with trauma limited to the mandible. CBCT imaging with three-dimensional reconstruction should be used to evaluate patients with known or suspected fractures of the mandibular condyle.

#### Rationale

Panoramic radiography can be used as an initial examination for evaluating mandibular fractures, including fractures of the condylar region. For mandibular fractures in general, the sensitivity of CT imaging (100%) is much higher than panoramic imaging [56]. CT better depicts fragment comminution and displacement. Notably, 50% of patients have more than one fracture in the mandible, and condylar fractures often occur with fractures at the mandibular symphysis and parasymphysis. The sensitivity of panoramic imaging for detection of condylar fractures (70%), in particular high condylar fractures, is much lower than CT imaging (92%) [57].

Although nondisplaced fractures can be difficult to detect on a panoramic radiograph, a discrepancy in the vertical dimension of the ramus with increased density in the area of the fracture can be a reliable diagnostic feature. Cross-sectional imaging, preferably with CBCT, and three-dimensional reconstruction of the fractured region is the imaging modality of choice.

To evaluate a patient with acute intra- and extracapsular trauma, T1- and T2WI may be prescribed to assess the integrity and position of the articular disc and the capsular attachments and to detect fluid accumulation and hemarthrosis [58].

## Recommendation for Assessment of Cysts and Neoplasms

Cysts and neoplasms of the TMJ region are uncommon [59] but may be the causative disease underlying a patient’s TMD symptoms. Clinical manifestations of progressive changes in occlusion and facial asymmetry are suggestive of morphologic changes in the condyle. Osteochondroma is one of the most common benign tumors of bone, but it is relatively rare in the maxillofacial region [60]. This condition is characterized by a slow-growing, osseocartilaginous exostosis with a defined periphery causing progressive deformity of the condyle. Other benign neoplasms of the TMJ region that share radiologic features with osteochondroma are osteoma, chondroma, and osteoblastoma. Malignant neoplasms, such as osteosarcoma and chondrosarcoma, are rare in the TMJ region [61]. Documented cases of metastasis to the TMJ are sparse [62, 63, 64, 65, 66]. Metastatic lesions may be lytic or sclerotic and are often expansile, with an infiltrative periphery causing cortical thinning, perforation, and expansion into adjacent soft tissues [67]. Periosteal reactions often display the characteristic “sun ray” or spiculated appearance. In general, metastases to the oral cavity are uncommon, accounting for 1% of all oral malignancies.

### Recommendation 6

CT imaging should be used to evaluate patients with known or suspected cysts and benign tumors in TMJ regions.

### Recommendation 7

CT imaging/MRI should be used to evaluate patients with known or suspected malignant tumors in TMJ regions.

#### Rationale

Imaging provides noninvasive assessment of TMJ morphology to detect cystic and neoplastic entities. The goal of CT imaging is to provide information on the nature, extent, and location of the abnormality in order to guide surgical management [68]. Histologic evaluation provides a definitive diagnosis.

The sensitivity and specificity values of MDCT for detection of bony abnormalities are 70% and 100%, respectively; with CBCT, these values are 80% and 100% [69]. The overall accuracy of CBCT and helical CT is 90% and 86%, respectively. Thus, CBCT and MDCT are equivalent for diagnostic evaluation of osseous abnormalities of the mandibular condyle. However, a critical assessment for potentially malignant lesions is extension into the adjacent soft tissues, an assessment that is not provided by CBCT. The presence of soft tissue involvement and its extent are important in making decisions for the adequate management of malignant lesions. Contrast-enhanced CT facilitates detection of these features [70]. MRI provides superior soft tissue resolution and may be performed in addition to, or in lieu of, CT imaging. General practice parameters for performance of these imaging studies are described elsewhere [71, 72].

Additional nuclear medicine imaging with single photon emission computed tomography (SPECT) or [18] F-fluorodeoxyglucose positron emission tomography (PET), typically fused with CT imaging (SPECT/CT and PET/CT), is often valuable in select patients with benign and malignant neoplasias [73]. A full discussion of the principles and applications of these imaging modalities is beyond the scope of this paper.

## Risk-Benefit Assessment

Imaging assessment of the TMJ is a valuable and often necessary step to establish the diagnosis and direct management of patients presenting with TMDs. The selection of imaging modality should consider the accuracy and adequacy of the imaging for the diagnostic task and its potential to provide information that contributes to diagnosis and management.

In addition to providing a diagnostic benefit, the risks associated with the imaging procedure should be reasonably low. The risks associated with imaging procedures recommended in this position statement are summarized here:

• Radiation detriment from conventional twodimensional imaging with intraoral and panoramic radiology is minimal.

• CBCT imaging is a low-dose CT imaging procedure, and the anticipated effective dose from typical protocols is < 150 mSv [74].

• Maxillofacial MDCT imaging delivers a higher dose compared to CBCT, with an anticipated effective dose increasing to approximately 1 mSv [75]. Nevertheless, maxillofacial MDCT delivers a lower dose compared to MDCT imaging of other organ sites.

• MRI does not use ionizing radiation; thus, there are no radiation-associated cancer risks.

Table 1 summarizes the applications of imaging for the diagnosis and management of conditions associated with TMDs. By applying the guidance provided in this document, clinicians can effectively design radiologic studies in which the benefits far outweigh the risks of radiation exposure.

**Table 1.** Application of Imaging for Diagnosis and Management of Conditions Associated with TMDs.

	Odontogenic diseases	Arthritic diseases	Developmental disorders	Internal derangement	Traumatic damage	Cysts/benign neoplasms	Malignant neoplasms	Radiation exposure*
Intraoral	++							⏭
Panoramic	+	+	+		+	+	+	⏭
CBCT (limited FOV)	++							⏭
CBCT (both arches)	+	++	++		++	++	+	⏭⏭
MDCT (maxillofacial)		++	++		++	++	++	⏭⏭
MRI (TMJ)		+	+	++	+	+	++	

*Relative radiation level designations as categorized by the American College of Radiology [76]. +Limited assessment. ++Detailed assessment.

## Conclusions

The evidence-based recommendations in this position statement will provide clinicians with guidance for appropriate and effective imaging of the TMJ.
